# A role for solvents in the toxicity of agricultural organophosphorus pesticides

**DOI:** 10.1016/j.tox.2012.02.005

**Published:** 2012-04-11

**Authors:** Michael Eddleston, Jonathan M. Street, Ian Self, Adrian Thompson, Tim King, Nicola Williams, Gregorio Naredo, Kosala Dissanayake, Ly-Mee Yu, Franz Worek, Harald John, Sionagh Smith, Horst Thiermann, John B. Harris, R. Eddie Clutton

**Affiliations:** a Clinical Pharmacology Unit, University/BHF Centre for Cardiovascular Science, University of Edinburgh, UK; b National Poisons Information Service - Edinburgh, Royal Infirmary, Edinburgh, UK; c Department of Anaesthesia, Royal (Dick) School of Veterinary Sciences, University of Edinburgh, UK; d Veterinary Pathology Unit, Royal (Dick) School of Veterinary Sciences, University of Edinburgh, UK; e Roslin Institute, University of Edinburgh, UK; f Centre for Statistics in Medicine, Oxford, UK; g Endocrinology Unit, University/BHF Centre for Cardiovascular Science, University of Edinburgh, UK; h Bundeswehr Institute of Pharmacology and Toxicology, Munich, Germany; i Medical Toxicology Centre and Institute of Neuroscience, Newcastle University, Newcastle upon Tyne, UK

**Keywords:** Suicide, Organophosphorus insecticides, Solvents, Cyclohexanone

## Abstract

Organophosphorus (OP) insecticide self-poisoning is responsible for about one-quarter of global suicides. Treatment focuses on the fact that OP compounds inhibit acetylcholinesterase (AChE); however, AChE-reactivating drugs do not benefit poisoned humans. We therefore studied the role of solvent coformulants in OP toxicity in a novel minipig model of agricultural OP poisoning. Gottingen minipigs were orally poisoned with clinically relevant doses of agricultural emulsifiable concentrate (EC) dimethoate, dimethoate active ingredient (AI) alone, or solvents. Cardiorespiratory physiology and neuromuscular (NMJ) function, blood AChE activity, and arterial lactate concentration were monitored for 12 h to assess poisoning severity. Poisoning with agricultural dimethoate EC40, but not saline, caused respiratory arrest within 30 min, severe distributive shock and NMJ dysfunction, that was similar to human poisoning. Mean arterial lactate rose to 15.6 [SD 2.8] mM in poisoned pigs compared to 1.4 [0.4] in controls. Moderate toxicity resulted from poisoning with dimethoate AI alone, or the major solvent cyclohexanone. Combining dimethoate with cyclohexanone reproduced severe poisoning characteristic of agricultural dimethoate EC poisoning. A formulation without cyclohexanone showed less mammalian toxicity. These results indicate that solvents play a crucial role in dimethoate toxicity. Regulatory assessment of pesticide toxicity should include solvents as well as the AIs which currently dominate the assessment. Reformulation of OP insecticides to ensure that the agricultural product has lower mammalian toxicity could result in fewer deaths after suicidal ingestion and rapidly reduce global suicide rates.

## 1 Introduction

Pesticides are used extensively in tropical agriculture to increase crop yield (World Health Organization, 1990). However, this use has a cost: pesticide self-poisoning is a major public health problem (Jeyaratnam, 1990; Eddleston and Phillips, 2004), killing at least 250–370,000 people every year (Gunnell et al., 2007a). Organophosphorus (OP) insecticides, acting as acetylcholinesterase (AChE) inhibitors, are the most important, being responsible for more than 2/3 of deaths due to their high toxicity and widespread use (Eddleston, 2000). Medical treatment is difficult, with case fatality often over 20% (Eddleston, 2000). We recently found that the specific antidote, the AChE reactivator pralidoxime, offers little benefit to patients severely poisoned with Environmental Protection Agency (EPA)/World Health Organization (WHO) Class II ‘moderately toxic’ OP insecticides (Eddleston et al., 2009a; Buckley et al., 2011). This suggests that other components of the agricultural OP formulations might be necessary for acute toxicity.

Although toxicity from coformulants is recognised for glyphosate herbicides (Bradberry et al., 2004), their role in the acute mammalian toxicity of the emulsifiable concentrate (EC) insecticide formulations used in agriculture and ingested in self-harm has been explored only once (Casida and Sanderson, 1961) and then apparently forgotten. Medical textbooks do not consider coformulants to be a clinical issue in OP insecticide poisoning. Of note, coformulants are usually present to improve the agricultural usability of the insecticide, not for their insecticidal activity.

To explore the role of coformulants in OP insecticide poisoning, we developed a Gottingen minipig (Forster et al., 2010b) model of poisoning with dimethoate EC40, the agricultural formulation of dimethoate that contains 400 g/l dimethoate active ingredient [AI] as well as coformulants. We chose the Gottingen minipig because from histological, anatomical and physiological perspectives, it is much closer to humans than rodents (Matute-Bello et al., 2008; Svendsen, 2006). Furthermore, due to similarities to human biochemistry and drug elimination, the Gottingen minipig has become an increasingly important model species for pharmacological and toxicological studies (Soucek et al., 2001; Svendsen, 2006; Forster et al., 2010a). The large size of the species has several further advantages including: a longer, and more clinically relevant, time course of study for most diseases; repeated sampling of blood and of the gas exchanging regions of the lung using bronchoalveolar lavage; and the use of readily available clinical equipment to measure physiology and for imaging.

The EPA/WHO Class II ‘moderately toxic’ insecticide dimethoate is a major clinical problem (Eddleston et al., 2005) with a case fatality of 20.6% in one large prospective case series (Dawson et al., 2010); it is likely to become more widely used following the Food and Agriculture Organization (FAO)’s advice to withdraw the more toxic Class I OP pesticides from agricultural practice (Food and Agriculture Organization of the United Nations, 2002) and recent favourable reviews by the EPA and FAO (Food and Agricultural Organization, 2005; US Environmental Protection Agency Office of Pesticide Programs, 2008). The EC40 formulation contains cyclohexanone, xylene, and a surfactant, as well as dimethoate (Table 1). Human poisoning with dimethoate EC40 is characterised by respiratory failure, distributive shock, cardiovascular collapse, and neuromuscular dysfunction (Eddleston et al., 2005; Davies et al., 2008).

We aimed to determine whether the dimethoate AI alone was responsible for the mammalian toxicity of agricultural dimethoate EC40 or whether other components of the formulation were necessary.

## 2 Methods

The study was performed under Home Office Licence after institutional ethics review in 27 adult male Göttingen minpigs (Ellegaard Minipigs ApS, Dalmose, Denmark) with mean weight 20.1 (SD 3.3) kg. Animals were drug-naïve and barrier bred, and shown to be free of infections before shipment to Edinburgh. Animals were kept in pens with free access to food scattered in their bedding and water under the care of institutional veterinary surgeons. Food was withheld for one night before a study. The animals were treated in accordance with the Animals (Scientific Procedures) Act of 1986.

### 2.1 Study design

The study involved three experiments: a comparison of dimethoate EC40 poisoning with saline placebo, a comparison of dimethoate AI and/or cyclohexanone with the results of this previous study, and a study of the experimental dimethoate EC35 formulation. See Table 2 for numbers of animals in each group. Each study was carried out separately in an intensive care laboratory, starting between 07:00 and 08:00.

The individual animal was the experimental unit. Bias was minimised by randomly allocating animals to study groups using a random number list. Allocation could not be predicted before allocation; the study was an open study but the outcomes were robust and not likely to be affected by bias (Wood et al., 2008).

### 2.2 Anaesthesia

Pre-anaesthetic medication was intramuscular injections of ketamine (5 mg/kg) and midazolam (0.5 mg/kg). Anaesthesia was induced with 5% isoflurane [selected since the effect of this anaesthetic on AChE activity is well characterised (Dorandeu et al., 2007)] in oxygen delivered via facemask. The trachea was intubated and anaesthesia maintained to a clinically acceptable depth using isoflurane in oxygen delivered via a circle breathing system. Intermittent positive pressure ventilation (IPPV) was provided as necessary using a minute volume divider (Manley Pulmovent, Harlow, UK) adjusted to maintain normocapnia.

Inspired and expired carbon dioxide, oxygen and isoflurane concentrations were monitored. Heart rate, oesophageal and peripheral temperature, electrocardiogram, and percentage of saturated haemoglobin were recorded (Datex, USA). Temperature was maintained as close to physiological values as possible by the use of forced warm air blankets (Bair Hugger, Arizant UK) or heat pads and a high ambient temperature. Ten ml/kg/h lactated Ringer's solution was administered for the first 30 min after induction of anaesthesia and then at 5 ml/kg/h for the remainder of the study. Fluid administration was increased as necessary during the study to maintain urine output and raise the central venous pressure.

### 2.3 Instrumentation and monitoring

A central arterial catheter was placed surgically into the carotid artery for continuous arterial pressure monitoring. A central venous catheter was placed into the external jugular vein for infusion of drugs and monitoring of central venous pressure. The catheters were connected to a pressure manometer (Datex, USA) zeroed at the level of the base of the heart to give arterial and CVP pressure readings. Lithium dilution cardiac output (LiDCO, London, UK) was used to assess beat-to-beat cardiac output, arterial blood pressure, and systemic vascular resistance (SVR). A urine catheter was placed by mini-laparotomy; urine output was measured every 60–120 min. An orogastric tube was placed for poison gavage.

IPPV was withdrawn every 30 min to assess the pig's ability to breathe spontaneously. The time to return of spontaneous ventilation (SV) and the EtCO_2_ after 30 s of SV were recorded. IPPV was then re-imposed to help maintain cardiovascular stability.

Mechanomyography was established using the deep peroneal nerve/tibialis posterior nerve/muscle group. Train of four stimulations was applied at 2 Hz, at intervals greater than 10 s, as per standard protocols (Fuchs-Buder et al., 2009).

### 2.4 Experimental protocol

After arterial catheter insertion, 60 min was allowed to pass before poisoning during which time baseline observations were recorded. Minipigs were randomly allocated to each group. Pigs were administered 2.5 ml/kg of dimethoate 40% emulsifiable concentrate (EC40; BASF SE, Ludwigs-hafen, Germany), 2.5 ml/kg saline placebo, 1 g/kg dimethoate AI (BASF), 1 g/kg cyclohexanone (Fluka-Sigma, Poole, UK), 1 g/kg xylene (Fluka-Sigma, Poole, UK), 1 g/kg dimethoate AI dissolved in cyclohexanone (1 ml/kg dose of cyclohexanone), or 2.5 ml/kg of dimethoate 40% emulsifiable concentrate lacking cyclohexanone (EC35; Cheminova A/S, Harboøre, Denmark), by gavage all followed by 60 ml of water. The quantity of each compound in each study represented the quantity present in a 2.5 ml/kg dose of agricultural dimethoate EC40. This allowed the results of each study to be compared with the original dimethoate EC40 study.

The initial dose of dimethoate EC40 was selected as being towards the middle range of the estimated dose in human self-poisoning (bottle sizes 100–400 ml (Eddleston et al., 2005), mean weight of self-poisoned patients 50 kg (Eddleston et al., 2000); likely dose range 0.1 to 8 ml/kg). Dose response studies with a 50% reduction in dimethoate EC40 dose caused mild poisoning that did not require high doses of noradrenaline (Eddleston et al., manuscript in preparation). The severe poisoning elicited by 2.5 ml/kg dimethoate EC40 allowed the components of the toxicity to be studied.

Noradrenaline was administered to maintain a MAP >55 mmHg, with a target MAP of 65 mmHg. Two hours post-dimethoate (EC or AI) or saline administration, a bolus of pralidoxime chloride (8 mg/kg) was given over 30 min followed by an infusion of 3.5 mg/kg/h until the end of the study. Atropine was administered as required to control muscarinic features. The study was ended by euthanasia using pentobarbital or anaesthetic overdose after 12 h.

### 2.5 Measurements

Cardiovascular data were collected 30 and 10 min before poisoning and 15 min intervals thereafter using LiDCO. Arterial blood samples were taken at −40, −10, and 30 min, and then every hour, and lactate analysed using an i-STAT (Abbott, NJ, USA). Analyses for red cell AChE activity were performed as previously described (Worek et al., 1999; Eddleston et al., 2005). Dimethoate and its active metabolite omethoate were detected by LC-ESI-MS/MS and FI-ESIMS/MS (Eddleston et al., 2005; John et al., 2010).

Cyclohexanone and cyclohexanol were quantified using a Thermo Scientific Trace gas chromato-graph fitted with an AS2000 autosampler and a flame ionisation detector. Plasma samples were prepared by thawing from −80 °C at room temperature, then 1 ml aliquots were spun in a micro-centrifuge for 5 min at 10,000 rpm to pellet any solid matter. 200 μl of supernatant was added to an autosampler vial containing 20 μl of 2 g/100 ml iso-amyl alcohol (internal standard) in water. One μl volumes of this mixture were injected and analysed using a HP-Innowax 30 m × 0.53 mm × 1 μm film thickness capillary column and the following conditions: injector temperature 240 °C, split ratio 6:1, carrier gas (helium) flow rate 1.8 ml/min, oven temperature programmed between 80 and 200 °C (2 min at 80 °C, then 15 °C/min increase to 200 °C); detector temperature 270 °C with hydrogen and air flow rates of 35 and 350 ml/min, respectively. Cyclohexanol, cyclohexanone and ethanol were quantified using an internal standard method with calibration over the range 0–10 mM.

### 2.6 Outcomes

The primary outcome for the study was arterial lactate concentration over the 12 h of the study; secondary outcomes were SVR, noradrenaline requirements, and red cell acetylcholinesterase activity over the 12 h of the study.

### 2.7 Power calculation

No data was available for calculating sample sizes before the study started. Groups of around five pigs were selected for the first study. Our choice of subsequent sample size was based on the experience from our first experiment and on minimising the use of animals.

### 2.8 Statistical analysis

We did primary data analysis in Prism 5.0 (GraphPad, San Diego, CA). All animals were included in the analysis. Pig weights were summarised with mean and SD; clinical and biochemical outcomes were summarised with mean and SEM. Due to the small number of animals, and our aim to include as much data in the analysis as possible, we compared the area under the curve for the outcomes of different minipig groups. All groups were compared using a Kruskal–Wallis test; if significant, we then performed pairwise comparisons with a non-parametric Mann–Whitney test. *P*-values obtained from the pairwise comparisons were adjusted for multiple comparisons using the FDR method (Benjamini and Hochberg, 1995). This was performed using the R Software Package version 2.14. Statistical significance was accepted at *P* < 0.05 for all tests.

## 3 Results

### 3.1 Agricultural dimethoate causes severe toxicity

**Fig. 1:**
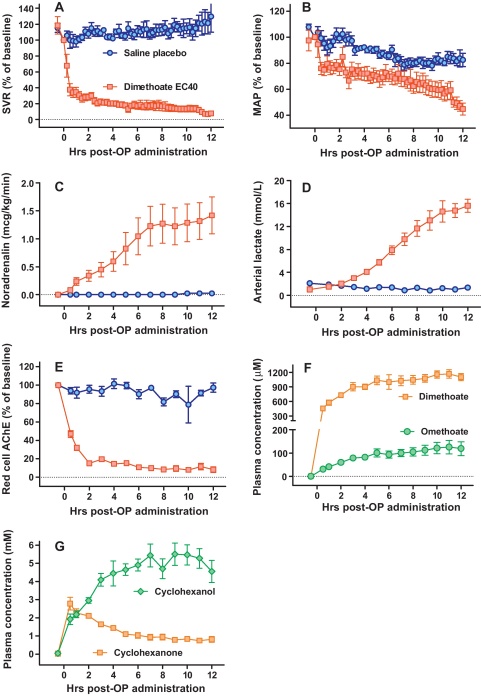
Cardiovascular and biochemical consequences of oral poisoning with dimethoate EC40 pesticide in Gottingen minipigs. (A) Systemic vascular resistance, (B) mean arterial pressure, (C) noradrenaline requirements, (D) arterial lactate concentration, and (E) red cell AChE activity in dimethoate EC40 (red squares) and saline placebo (blue rings) poisoned minipigs. Pralidoxime was administered from 2 h post poison administration. (F) Plasma concentration of dimethoate (brown squares), and its active metabolite omethoate (green circles), and of (G) the main solvent cyclohexanone (brown squares), and its metabolite cyclohexanol (green diamond), in minipigs poisoned with dimethoate EC40. The graphs show data over the 12 h of the study; mean ± SEM, *n* = 5–7 pigs. (For interpretation of the references to color in this figure legend, the reader is referred to the web version of the article.)

**Fig. 2:**
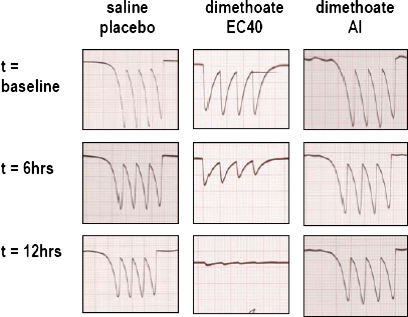
Neuromuscular function in poisoned minipigs. Neuromuscular function shown by mechanomyography in pigs poisoned with saline placebo, dimethoate EC40, and dimethoate AI at baseline. NMJ dysfunction is clearly visible at 6 h in pigs poisoned by dimethoate EC40; NMJ function was normal throughout the study in the other pigs. Four stimuli were produced at 2 Hz as per standard TOF methodology (Fuchs-Buder et al., 2009).

Dimethoate EC40 2.5 ml/kg (containing 1 g/kg active ingredient [AI] dimethoate) given by gavage resulted in respiratory arrest within 30 min; spontaneous breathing did not recur during the 12 h study. Noradrenaline (NA) was soon required to maintain the mean arterial pressure (MAP) above 55 mmHg (target 65 mmHg) due to a rapid fall in systemic vascular resistance (SVR; Fig. 1). The SVR and MAP continued to fall, requiring increasing doses of NA; there was a concurrent rise in heart rate, stroke volume, and cardiac output (data not shown), as well as arterial blood lactate (to 15.6 [SD 2.8] mmol/l at 12 h). Administration of saline placebo produced only minor changes in SVR and MAP, and no rise in arterial blood lactate (1.4 [SD 0.8] mmol/l at 12 h, *P* < 0.0268; Fig. 1, Table 2). Monitoring of neuromuscular junction (NMJ) function by mechanomyography (MMG) showed gradual dysfunction in dimethoate EC40 poisoned pigs (Fig. 2).

Pralidoxime chloride was administered at 2 h post-poisoning; examination of red cell AChE activity showed little reactivation (Fig. 1E). In addition, red cell AChE assays showed that the respiratory failure and the initial distributive shock (both of which occurred within 30 min of ingestion) occurred before AChE activity had fallen by more than 70%. This suggests that AChE inhibition alone is not responsible for clinical toxicity, since human studies indicate that >70% inhibition is required for clinical illness (Thiermann et al., 2005).

### 3.2 Dimethoate AI and cyclohexanone reproduced agricultural dimethoate's toxicity

**Fig. 3:**
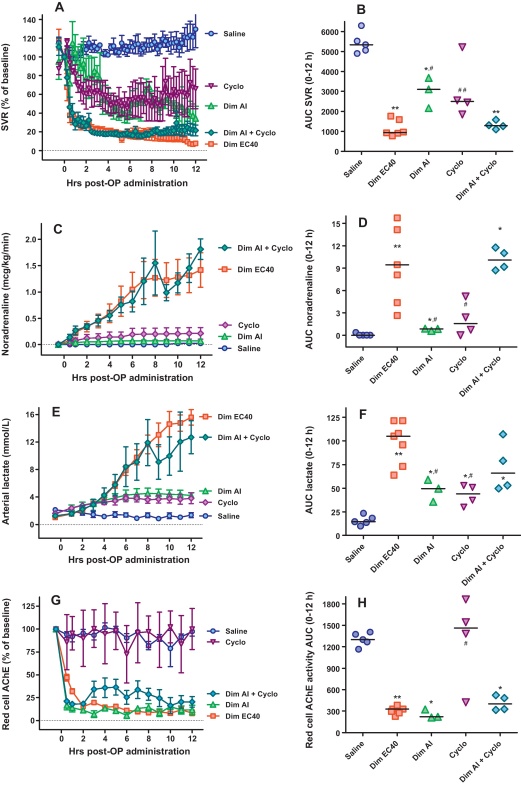
Cardiovascular and biochemical consequences of oral poisoning with dimethoate EC40 pesticide, dimethoate AI, and/or cyclohexanone in Gottingen minipigs. (A and B) systemic vascular resistance (SVR), (C and D) noradrenaline requirements, (E and F) arterial lactate concentration, and (G and H) red cell AChE activity in groups of pigs poisoned with saline placebo (blue circles), dimethoate EC40 (red squares), dimethoate AI (green triangles), cyclohexanone (purple diamonds) and dimethoate AI plus cyclohexanone (blue-green diamonds). The saline and dimethoate EC40 data from Fig. 1 are repeated here to contrast the effect of different poisons. The graphs show data over the 12 h of the study; mean ± SEM, *n* = 3–6 pigs. The dot plots show the AUC and median for each group. Comparison of all groups with a Kruskal–Wallis test indicated significant differences for all four variables. The results of pairwise comparisons using the Mann–Whitney test are given in the figures (*P < 0.05 and **P < 0.01 compared to saline placebo; ^#^*P* < 0.05 and ^##^*P* < 0.01 compared to dimethoate EC40). *Abbreviations*: cyclo, cyclohexanone; dim, dimethoate. (For interpretation of the references to color in this figure legend, the reader is referred to the web version of the article.)

To test whether coformulants might be responsible for toxicity, we then prepared solutions of (i) technical grade dimethoate AI alone, (ii) cyclohexanone alone, (iii) xylene alone (data not shown), and (iv) dimethoate AI and cyclohexanone together. Each compound was given at a dose of 1 g/kg, reproducing their dose in the EC40 formulation, except for xylene (8-fold larger quantity than present in dimethoate EC40) (Table 1). Pralidoxime was given to pigs receiving dimethoate AI. Pigs receiving cyclohexanone alone or dimethoate AI alone had modest falls in SVR and MAP that did not require large doses of NA or result in a marked rise in arterial lactate (Fig. 3A–F). Xylene showed no toxicity (data not shown). However, pigs given dimethoate AI and cyclohexanone together showed identical toxicity to dimethoate EC40 with rapid respiratory arrest, severe distributive shock, marked rise in arterial lactate (Fig. 3A–F, Table 2), and NMJ dysfunction.

**Fig. 4:**
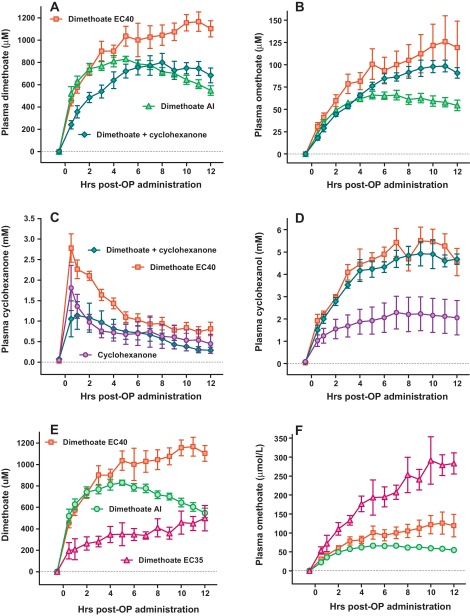
Pharmacokinetics of dimethoate and cyclohexanone, and their metabolites, in poisoned minipigs. Plasma (A) dimethoate and (B) omethoate concentrations in pigs poisoned with dimethoate EC40 (red squares), dimethoate AI (green triangles) and dimethoate AI and cyclohexanone together (blue-green diamonds). Plasma (C) cyclohexanone and (D) cyclohexanol concentrations in pigs poisoned with dimethoate EC40 (red squares), cyclohexanone (purple circles) and dimethoate AI and cyclohexanone together (blue-green diamonds). Plasma (E) dimethoate and (F) omethoate in pigs poisoned by dimethoate EC35 (purple triangles) compared to dimethoate EC40 (red squares) and dimethoate AI (green circles). The graphs show data over the 12 h of the study; mean ± SEM, *n* = 3–6 pigs. (For interpretation of the references to color in this figure legend, the reader is referred to the web version of the article.)

The modest effects of dimethoate AI or cyclohexanone alone was not due to reduced absorption. Analysis of red cell AChE activity showed similar inhibition with dimethoate AI and EC40 (Fig. 3G and H). There was no pralidoxime-induced reactivation of AChE inhibited by dimethoate AI. The plasma concentration for dimethoate and its active metabolite, omethoate, were similar during the first 4 h post-poisoning (Fig. 4) when marked differences in clinical syndrome were apparent. Similarly, concentrations of plasma cyclohexanone and its metabolite, cyclohexanol, soon after poisoning were similar in pigs receiving the three formulations containing cyclohexanone (Fig. 4). However, plasma concentrations of cyclohexanol were 3-fold higher at later time points in pigs receiving dimethoate EC40 or dimethoate AI + cyclohexanone than cyclohexanone alone.

### 3.3 Agricultural dimethoate without cyclohexanone is less toxic

**Fig. 5:**
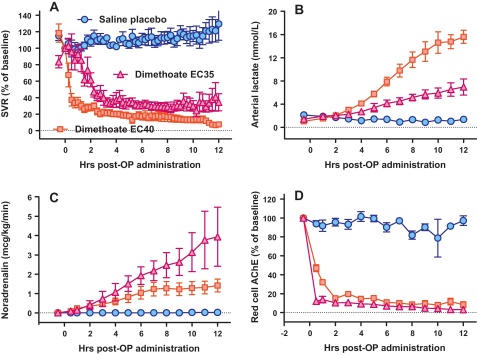
Cardiovascular and biochemical consequences of oral poisoning with dimethoate EC35 pesticide versus saline placebo and dimethoate EC40 in Gottingen minipigs. (A) Systemic vascular resistance, (B) noradrenaline requirements, (C) arterial lactate concentration, and (D) red cell AChE activity in groups of pigs poisoned with saline placebo (blue circles), dimethoate EC40 (red squares), or dimethoate EC35 (purple triangles). The saline and dimethoate EC40 data from Fig. 1 are repeated here to contrast with the effect of dimethoate EC35. The graphs show data over the 12 h of the study; mean ± SEM, *n* = 4–6 pigs. (For interpretation of the references to color in this figure legend, the reader is referred to the web version of the article.)

We then tested an experimental EC formulation (dimethoate EC35) that also contained 400 g/l dimethoate but no cyclohexanone. Administration of 2.5 ml/kg resulted in no respiratory arrest or NMJ dysfunction. The cardiovascular toxicity was similar to dimethoate EC40, with requirements for large doses of NA (Fig. 5C; Table 2). However, the rise in mean arterial lactate (to 7.0 [SD2.8] mmol/l at 12 h; Fig. 5B, Table 2) occurred more slowly. Of note, despite the less severe respiratory toxicity and smaller rise in lactate noted in this model, red cell AChE inhibition was more severe (Fig. 5D; Table 2) and omethoate plasma concentration greater than following poisoning with the EC40 formulation (Fig. 4), again suggesting that factors other than the OP determine toxicity.

## 4 Discussion

In this work, we developed a model of OP pesticide poisoning that is highly relevant to human self-poisoning. We used a relevant dose of formulated agricultural dimethoate, given by a relevant route, to a species with many physiological and metabolic similarities to humans, treated in a similar way to human patients. The severe cardiovascular shock and neuromuscular dysfunction, and lack of effect of pralidoxime, that resulted was very similar to human dimethoate poisoning. Treatment of poisoned patients is difficult with a high case fatality for severely poisoned patients. Fifty years of animal research has not brought a single new treatment into clinical use (Buckley et al., 2004). This clinically relevant model may be useful for testing novel antidotes.

We found that the severe toxicity was not due only to the dimethoate AI itself. Instead, the cyclohexanone solvent was required for toxicity – its absence resulted in no neuromuscular toxicity and markedly attenuated cardiotoxicity. Poisoning with an experimental formulation of agricultural dimethoate that lacked cyclohexanone produced less toxicity.

These results clearly indicate that the toxicity of the agricultural dimethoate preparations ingested for self-harm in rural Asia is due to both the dimethoate AI and its major solvent cyclohexanone. Each compound alone is unable to cause severe toxicity. This finding has profound public health and clinical implications.

OP insecticides have been formulated to enhance their agricultural efficacy and safety, not to make them safer for human self-poisoning. This might seem reasonable, since the bottle label clearly states that the insecticide should not be drunk. However, farming in the developing world is stressful, and self-harm with insecticides (Eddleston and Phillips, 2004), whether due to crop failure, indebtness, alcoholism, or simple social stresses, must be thought of as an occupational hazard of farming practices in which widespread and easy access to pesticides is encouraged by government and industry. In this case, reformulation of pesticides to make them less toxic to humans should be a priority. The introduction of less toxic OP pesticides into agricultural practice should markedly reduce suicide rates, as shown by Sri Lanka's experience in the mid-1990s when method substitution was minimal (Gunnell et al., 2007b; de Silva et al., 2012).

Unfortunately, risk assessment of pesticide toxicity concentrates on the active ingredient, not on the other constituents of the formulated pesticides, as shown by recent FAO and EPA assessments performed on dimethoate (US Environmental Protection Agency Office of Pesticide Programs, 2008; Food and Agricultural Organization, 2005). For formulated products, toxicity information usually only consists of acute toxicity data generated in rodents for the purpose of classification and labelling. There is relatively little knowledge about the comparative toxicity of differently formulated pesticides or the role of coformulants in overall acute toxicity.

The importance of solvents in dimethoate toxicity may explain in part the inability of pralidoxime to markedly improve outcome for patients poisoned with WHO Class II OP insecticides (Eddleston et al., 2009a; Buckley et al., 2011). There is currently no specific antidote for solvents; oximes may be addressing only part of the toxicity. Namba showed clearly in the 1950s that pralidoxime benefited patients unintentionally poisoned with the more toxic WHO Class I OP insecticides such as parathion (Namba and Hiraki, 1958; Namba et al., 1959). This may be due to lower level occupational exposure; however, it may also be because the quantity of solvent present in a lethal dose of parathion is markedly less than in a ‘moderately toxic’ WHO Class II OP, reducing the importance of coformulants. This situation is also likely to be quite different after poisoning with OP nerve agents (e.g. sarin) in which there are no solvents and the onset is much faster, making it likely that AChE inhibition is responsible for all toxic features (Maxwell et al., 2006).

Toxicokinetic and dynamic studies indicated that the differences were not due to variation in absorption alone. Red cell AChE activity in pigs poisoned with dimethoate EC40 and dimethoate AI were identical, despite very different poisoning severity. This discrepancy raises questions about the usefulness of this biomarker in OP pesticide poisoning (Eddleston et al., 2009a,b; Eyer et al., 2010).

Plasma dimethoate and omethoate concentrations were similar in the first few hours after poisoning with dimethoate EC40, dimethoate AI, and dimethoate AI + cyclohexanone, when differences in toxicity were apparent. The dimethoate and omethoate concentrations after poisoning with dimethoate AI then decreased. The dimethoate concentration after poisoning with the new dimethoate EC formulation was markedly less than with the other formulations; however, the omethoate concentration was significantly higher and red cell AChE more inhibited, suggesting again that pesticide toxicokinetic differences were not the basis for the differences in toxicity.

Plasma cyclohexanol concentrations were substantially lower after poisoning with cyclohexanone alone compared to dimethoate EC40 or dimethoate + cyclohexanone. Plasma cyclohexanone concentrations were also lower after cyclohexanone compared to dimethoate EC40 but less so than its metabolite. These differences suggest that the presence of dimethoate alters metabolism of the solvent; it is known that dimethoate induces cytochrome P450 activity and its own metabolism (Buratti and Testai, 2007). There was little evidence for dimethoate increasing the absorption of cyclohexanone.

The mechanism for the effect of cyclohexanone on dimethoate toxicity is unclear. Both dimethoate AI and cyclohexanone caused a fall in systemic vascular resistance; it is possible that their effects are additive. Alternatively, the solvent may alter the distribution of the dimethoate and thereby alter toxicity. Further studies are required to address this point.

We used arterial lactate concentration as a marker of global toxicity. Its substantial increase in pigs poisoned with dimethoate and cyclohexanone probably represents a combination of tissue hypoxia, hepatic dysfunction reducing lactate clearance, and catecholamine-induced changes in muscle metabolism.

The main limitation of this study is that it was performed in anaesthetised minipigs and not humans. An anaesthetised minipig is clearly different to self-poisoned humans and we cannot be sure that the results are a “true reflection” of the human situation. However, such a study is not possible in humans and pigs have multiple similarities to humans (McAnulty et al., 2011), increasing the relevance of these results. The great majority of previous research studies for investigation of OP antidotes have been done in rodents. These studies have limited relevance to acute human toxicity as seen in self-poisoning: rodents metabolize xenobiotics differently to humans (Martignoni et al., 2006; Tang et al., 2001). In addition, the OP compound has usually been given by an irrelevant route (e.g. intravenous), as an unformulated AI, and the rodent has not been treated with supportive care (ventilation, vasopressors) as occurs for humans. Our model is more relevant to human poisoning, with the pesticide given by the correct route and formulation to a species that has physiological and biochemical similarities to human, receiving typical supportive care. This relevance is apparent in the similarity of the poisoning seen in these minipigs and poisoned humans, with the same clinical syndrome, AChE inhibition, and response to pralidoxime (Davies et al., 2008; Eddleston et al., 2005). Although in vitro studies indicate that pig AChE inhibited by highly toxic OP nerve agents is less well activated by oximes than similarly inhibited human AChE (Aurbek et al., 2006; Worek et al., 2008), this is moot here since human AChE is not reactivated by pralidoxime – the same situation as we found in our pig model. Use of such a model in future animal studies will reduce the number of animals used (3Rs).

Due to welfare issues, the animals were anaesthetised with isoflurane for the study. Although the use of anaesthesia is another limitation, all arms of the study had identical anaesthesia and this could not explain the differences seen. Isoflurane anaesthesia was selected since it has a consistent and mild (about 10%) inhibitory effect on AChE activity (Dorandeu et al., 2007). The animals were anaesthetised for about 1.5 h before poison administration. Differences between arms of the study were apparent within 30 min, making it unlikely in this time frame that induction of CYP enzymes involved in the metabolism of OPs could be involved in the differences seen.

In conclusion, this study indicates that dimethoate AI is not solely responsible for toxicity in the pig. Instead, coformulants are an important element of OP toxicity; therefore their toxicity should be considered by manufacturers, regulators and clinicians. Further studies are required to determine the generality of this finding and how formulations can be changed to improve human safety without reducing agricultural efficacy.

## Contributions

ME designed the study, established the minipig model, performed the studies and analysis, and wrote the first draft with input from all authors. JMS, IS, TK, KD performed the studies. AT, FW, HJ, SS and HT performed the biochemical analyses. ME did the statistical analysis with NW and LMY. GN prepared the chemicals for administration. JBH and REC developed the minipig model with ME and performed the studies. All authors read and approved the final version of the manuscript before submission.

## Role of the funding source

The funders had no role in study design, data collection, data analysis, data interpretation, or writing of the report. The corresponding author had full access to all the data in the study and had final responsibility for the decision to submit for publication.

## Funding

This work was supported by the Wellcome Trust (grants GR063560, GR085979, GR090886), and the BUPA Foundation (BUPA medical research prize). ME is a Scottish Senior Clinical Research Fellow (Scottish Chief Scientist Office/Scottish Funding Council) and Lister Research Prize Fellow (Lister Institute for Preventative Medicine).

## Conflict of interest

ME has received an unrestricted research grant for minipig studies from Cheminova. Cheminova did not fund this study and had no role in the analysis, write up, or other aspects of the research. All other authors declare they have no competing financial interests.

## Figures and Tables

**Table 1:** Constituents of the dimethoate EC40 (BASF) formulation used in agriculture.

Constituent	CAS number	Role	Amount (g/l)	Toxicity (rat oral LD50, mg/kg)	Number of rat oral LD50/kg in 2.5 ml/kg
Dimethoate	60-51-5	Active ingredient	40	250 (World Health Organization, 2010)	4
Cyclohexanone	108-94-1	Solvent	40	1620 (Smyth et al., 1969)	0.62
Xylene	1330-20-7	Solvent	5	∼5000 (International Programme on Chemical Safety, 1997)	0.025
*Wettol*		Surfactant	NK	Low	NK

The surfactant, *Wettol*, is not described on the Product Safety Information Sheet and is therefore likely to have been non-hazardous in rat studies. It is a propriety compound and does not have a CAS number. *Abbreviations*: CAS, Chemical Abstract Service; NK, not known.

**Table 2:** Comparisons of lactate concentration, SVR, noradrenaline requirements, and red cell AChE between study groups.

Group	AUC (1–12 h) Mean (SEM)	Comparison with Saline control	Comparison with Dimethoate EC40	AUC (1–12 h) Mean (SEM)	Comparison with Saline control	Comparison with Dimethoate EC40
	Arterial lactate			SVR		
Saline control (*n* = 5)	16.0 (2.3)	N/A	*P* = 0.0268	5423 (244)	N/A	*P* = 0.0358
Dimethoate EC40 (*n* = 7)	98.4 (8.5)	*P* = 0.0268	N/A	1151 (169)	*P* = 0.0358	N/A
Dimethoate AI (*n* = 3)	48.0 (6.8)	*P* = 0.0402	*P* = 0.0285	2979 (442)	*P* = 0.0490	*P* = 0.0428
Cyclohexanone (*n* = 4)	42.7 (5.4)	*P* = 0.0268	*P* = 0.0268	3013 (748)	*P* = 0.0714	*P* = 0.0358
Dim AI + cyclo (*n* = 4)	72.2 (13.3)	*P* = 0.0268	*P* = 0.1554	1314 (100)	*P* = 0.0358	*P* = 0.4762
Dimethoate EC35 (*n* = 4)	52.6 (5.9)	*P* = 0.0268	*P* = 0.0268	2176 (229)	*P* = 0.0358	*P* = 0.0490

Group	AUC (1–12 h) Mean (SEM)	Comparison with Saline control	Comparison with Dimethoate EC40	AUC (1–12 h) Mean (SEM)	Comparison with Saline control	Comparison with Dimethoate EC40
	Noradrenaline requirements			Red cell AChE activity		
Saline control	0.07 (0.1)	N/A	*P* = 0.0376	1303 (42)	N/A	*P* = 0.0183
Dimethoate EC40	11.3 (2.9)	*P* = 0.0376	N/A	310 (19)	*P* = 0.0183	N/A
Dimethoate AI	0.77 (0.1)	*P* = 0.0393	*P* = 0.0376	253 (35)	*P* = 0.0536	*P* = 0.1500
Cyclohexanone	2.09 (1.1)	*P* = 0.1043	*P* = 0.0393	1304 (310)	*P* = 0.4127	*P* = 0.0183
Dim AI + cyclo	10.2 (0.7)	*P* = 0.0376	*P* > 0.999	413 (53)	*P* = 0.0286	*P* = 0.3546
Dimethoate EC35	24.0 (6.6)	*P* = 0.0376	*P* = 0.1227	200 (6)	*P* = 0.0286	*P* = 0.0183

All groups were compared using a Kruskal–Wallis test; since this comparison was significant, we then performed pairwise comparisons with a non-parametric Mann–Whitney test with adjustment of multiple comparisons using the FDR method.
